# The implementation of long-lasting insecticidal bed nets has differential effects on the genetic structure of the African malaria vectors in the *Anopheles gambiae* complex in Dielmo, Senegal

**DOI:** 10.1186/s12936-017-1992-8

**Published:** 2017-08-15

**Authors:** Seynabou Sougoufara, Cheikh Sokhna, Nafissatou Diagne, Souleymane Doucouré, Pape MBacké Sembène, Myriam Harry

**Affiliations:** 1 URMITE (Unité de Recherche sur les Maladies Infectieuses et Tropicales Emergentes), UM63, CNRS 7278, IRD 198, INSERM 1095, IHU-Méditerranée Infection, Marseille, France; 2 Département de Biologie Animale, Faculté des Sciences et Techniques/Université Cheikh Anta Diop, Dakar, Senegal; 3 UMR EGCE (Évolution, Génomes, Comportement, Écologie) CNRS, IRD-Université Paris-Sud, IDEEV, Université Paris-Saclay, Gif-sur-Yvette Cedex, France

**Keywords:** Malaria, *An. gambiae*, *An. coluzzii* and *An. arabiensis*, LLINs, Genetic structure, Senegal

## Abstract

**Background:**

Mosquitoes belonging to the *Anopheles gambiae* complex are the main vectors of malaria in sub-Saharan Africa. Among these, *An. gambiae, Anopheles coluzzii* and *Anopheles arabiensis* are the most efficient vectors and are largely distributed in sympatric locations. However, these species present ecological and behavioural differences that impact their vectorial capacity and complicate vector-control efforts, mainly based on long-lasting insecticidal bed nets (LLINs) and indoor residual spraying (IRS). In this study, the genetic structure of these three species in a Senegalese village (Dielmo) was investigated using microsatellite data in samples collected in 2006 before implementation of LLINs, in 2008, when they were introduced, and in 2010, 2 years after the use of LLINs.

**Results:**

In this study 611 individuals were included, namely 136 *An. coluzzii,* 101 *An. gambiae*, 6 *An. coluzzii/An. gambiae* hybrids and 368 *An. arabiensis*. According to the species, the effect of the implementation of LLINs in Dielmo is differentiated. Populations of the sister species *An. coluzzii* and *An. gambiae* regularly experienced bottleneck events, but without significant inbreeding. The Fst values suggested in 2006 a breakdown of assortative mating resulting in hybrids, but the introduction of LLINs was followed by a decrease in the number of hybrids. This suggests a decrease in mating success of hybrids, ecological maladaptation, or a lesser probability of mating between species due to a decrease in *An. coluzzii* population size. By contrast, the introduction of LLINs has favoured the sibling species *An. arabiensis.* In this study, some spatial and temporal structuration between *An. arabiensis* populations were detected, especially in 2008, and the higher genetic diversity observed could result from a diversifying selection.

**Conclusions:**

This work demonstrates the complexity of the malaria context and shows the need to study the genetic structure of *Anopheles* populations to evaluate the effectiveness of vector-control tools and successful management of malaria vector control.

**Electronic supplementary material:**

The online version of this article (doi:10.1186/s12936-017-1992-8) contains supplementary material, which is available to authorized users.

## Background

Over the last decade, impressive progress has been made in controlling malaria vectors in sub-Saharan Africa mainly using long-lasting insecticidal bed nets (LLINs) and indoor residual spraying (IRS) [1]. Both tools have remarkably contributed to the decrease of malaria prevalence since 2000 [2] in sub-Saharan Africa, where the burden of malaria is heaviest [3]. Three species of the *Anopheles gambiae* complex, namely *Anopheles gambiae*, *Anopheles coluzzii* and *Anopheles arabiensis* are the primary vectors of malaria in this region [4]. Indeed, these species coexist in sympatry throughout their range and are closely associated with human habitats [5]. The two former species, *An. gambiae* and *An. coluzzii,* were previously identified as the two *An. gambiae* molecular forms, S and M, respectively [6]. Evidence of hybridization or introgression between these species raised questions about their taxonomic status [7–14]. The recognized sibling species *An. arabiensis* was never divided into chromosomal forms, although it is widely distributed in the Afrotropical region [15]. Behavioural differences that impact their vectorial capacity as malaria vectors have been recorded between these sibling species. Indeed, *An. gambiae* and *An. coluzzii* are highly anthropophilic and endophilic [5].

*Anopheles arabiensis* displays similar patterns, but presents a competitive advantage of being more zoophilic and exophilic [16]. These phenotypic differences highlight genetic heterogeneities within the *An. gambiae* complex that could affect the species habitat preference and their adaptive responses against malaria control tools. Several studies have largely reported selective pressure on malaria vectors after the widespread use of vector-control tools using insecticide compounds. The most obvious example is the selection and the emergence of insecticide resistance genes in the main vectors in sub-Saharan Africa [17–22]. Furthermore, when the LLINs are used, a selective advantage has been reported in *Anopheles* species that exhibit a tendency to feed outdoors, earlier in the evening, during diurnal hours and on non-human hosts [23–25]. Indeed, a previous study carried out in Nigeria and based on cytogenetic analyses has reported the presence of chromosomal inversions correlated with the indoor or outdoor biting preferences in the main vectors of the *An. gambiae* complex [26]. Furthermore, in Burkina Faso, in 2011, an outdoor-resting subgroup of the *An. gambiae* complex, named “Goundry”, genetically isolated from both the M (*An. coluzzii*) and S (*An. gambiae*) forms was found exclusively in peridomestic human habitats [27]. The authors suggest that this outdoor subgroup would be favoured by selective pressure of insecticide-treated bed nets and indoor residual spraying used in the area. Some studies showed the persistence of *An. arabiensis* following the implementation of insecticide-treated bed nets and its potential involvement in residual malaria transmission [23, 28]. These events could jeopardize the effectiveness of vector-control tools using insecticide compounds due to a non-uniform exposure of vectors to insecticide and worsen malaria transmission. Thus, in the context of scaling up malaria vector-control tools using insecticide-treated bed nets and indoor residual spraying, it is essential to know whether selective pressure affects the genetic structure of the main vectors of the *An. gambiae* complex relative to their habitat feeding place, a paramount factor in understanding the malaria epidemiology.

Concerning *An. gambiae* and *An. coluzzii,* previous investigations on the genetic differentiation of both species have reported divergent findings, even though the geographical scale, the pattern of genomic tools used and the genomic island analysed were not homologous [29–36]. Genetic differentiation between populations of the sibling species, *An. arabiensis* was reported according to geographical distance [37, 38]. But, in Tanzania, high levels of differentiation were reported within *An. arabiensis* sympatric samples [39]. Senegalese populations of the genetic structure of the *An. gambiae* species complex were investigated only in two *An. arabiensis* populations collected in Dielmo and Barkedji villages (250 km apart) [40] between which low but significant levels of genetic differentiation were found.

As in other African malaria-endemic regions, Senegal has adopted universal coverage of insecticide-treated bed nets in its strategic national program to accelerate efforts toward malaria pre-elimination. Thus, several studies have reported on the malaria transmission dynamics and the monitoring of entomological parameters on *Anophele*s vectors [28, 41, 42]. However, no information was available on the impact of insecticide-treated bed nets on the genetic structure of the main vectors of the *An. gambiae* complex.

This study focuses on the impact of long-lasting insecticidal treated bed nets on the genetic structure of *An. coluzzii* and *An. gambiae* species and their sibling species *An. arabiensis* through a spatiotemporal survey covering 3 years of entomological surveys. The occurrence of putative gene flows or hybridization between *An. coluzzii* and *An. gambiae* is also considered. The implication of the control strategy is discussed.

## Methods

### Study area and mosquito sampling

The study was conducted in Dielmo village (13°45′N, 16°25′W), where a longitudinal survey has been carried out since 1990 to identify all malaria episodes and to investigate the relationship between malaria parasites, human hosts and vectors [43]. The village is located 280 km Southeast of Dakar and about 15 km North of the Gambian border. In Dielmo, the rainy season occurs mostly from June to October. In July 2008, long-lasting insecticide-bed nets (LLINs) were distributed to all villagers. This was followed by a decrease to malaria incidence between August 2008 and August 2010 [44]. However, two episodic resurgences of malaria attacks were noted in adults and children aged up to 10 years old in 2010 and 2013 [44, 45].

In the village, since the establishment of the project, mosquitoes have been captured monthly using human landing catches (HLC) from indoor and outdoor habitations between 7 p.m. and 7 a.m. for three consecutive nights. All anopheline species collected were identified in the field using the morphological identification keys of Gilles and Coetzee [46] and the specimens were individually stored in Eppendorf tubes containing silica gel and then taken for further molecular analysis. In this study, mosquitoes were sampled during the rainy season in September and October 2006 (2 years before LLINs), in July 2008 (date of implementation of LLINs), September 2008 and September and October 2010 (2 years after LLINs were first used).

### DNA extraction and species identification

Genomic DNA from each mosquito was extracted using the kit NucleoSpin Tissue XS made by Machery-Nagel. DNA extracts were amplified by polymerase chain reaction (PCR) to discriminate species within the *An. gambiae* complex (Table 1) using the protocol of Wilkins et al. [47], with intentional mismatch primers recognising single nucleotide polymorphisms (SNPs) at the 3-prime end (IMP-PCR).

**Table 1:** Sampling and identification of mosquitoes

Protocol of identification	Years	Months	Location of capture	*An. coluzzii*	*An. gambiae*	Hybrids	*An. arabiensis*	Total
A (n = 611)	2006	September	Indoor	21	14	1	7	43
			Outdoor	44	4	0	17	65
		October	Indoor	10	6	3	6	25
			Outdoor	18	5	0	9	32
		Total		93	29	4 (3.2%)	39	165
	2008	July	Indoor	0	2	0	53	55
			Outdoor	1	0	0	82	83
		October	Indoor	14	3	1	35	53
			Outdoor	10	2	0	24	36
		Total		25	7	1 (3.03%)	194	227
	2010	September	Indoor	8	20	1	42	71
			Outdoor	7	13	0	31	51
		October	Indoor	1	16	0	19	36
			Outdoor	2	16	0	43	61
		Total		18	65	1 (1.2%)	135	219
	Total			136	101	6	368	611
B (n = 497)		*An coluzzii*		109	2	0	1	112 (97.32)
		*An. gambiae*		4	86	0	0	90 (95.55)
		*An. coluzzii/An. gambiae* hybrids		5	0	1	0	6 (16.66)
		*An. arabiensis*		0	1	0	288	289 (99.65)

A. Information on population size for each species identified each year, month and location of collection. The mosquitoes were identified according to Wilkins et al.’s protocol [47] (see “Methods”). B. A subset of individuals was also examined according to Santolamazza et al.’s protocol [49] (the percentage of congruent determination with the Wilkins et al.’s protocol is given in brackets)

In order to have a sufficient sample size for microsatellite analysis, i.e. no less than 15 individuals, some samples were pooled as follows (Table 2): *An. coluzzii* and *An. gambiae* samples were analysed according to the location of capture, indoor and outdoor in each year, and if necessary indoor and outdoor samples of each species were pooled per year. *Anopheles arabiensis* samples were analysed according to the location of capture in each month (July; September; October) except in 2006. The identified hybrids between *An. coluzzii* and *An. gambiae* (six individuals) were not included in the general microsatellite analysis in order to avoid bias in gene flow estimation, but were subjected to the assignment test.

**Table 2:** Sample codes and population sizes of *An. coluzzii*, *An. gambiae* and *An. arabiensis* samples analysed using microsatellites markers

Sample species	Months/year	Location of capture	Sample codes	Sample sizes
*An. coluzzii*	2006	Indoor	ACIND06	22
		Outdoor	ACOUT06	57
	2008	Indoor and outdoor	AC08	25
	2010	Indoor and outdoor	AC10	15
*An. gambiae*	2006	Indoor and outdoor	AG06	23
	2010	Indoor	AGIND10	32
		Outdoor	AGOUT10	27
*An. arabiensis*	Sept 2006	Outdoor	AROUTSEP06	15
	2008			
	July	Indoor	ARINDJUL08	44
		Outdoor	AROUTJUL08	72
	Sept	Indoor	ARINDSEP08	33
		Outdoor	AROUTSEP08	22
	2010			
	Sept	Indoor	ARINDSEP10	38
		Outdoor	AROUTSEP10	27
	Oct	Indoor	ARINDOCT10	19
		Outdoor	AROUTOCT10	39

In the sample codes the two first letters refer to the species name (AC: *An. coluzzii*; AG: *An. gambiae*; AR: *An. arabiensis*), the following three letters to the location of capture (IND: indoor; OUT: outdoor); and the number to the year of sampling (06: 2006; 08: 2008; 10: 2010). If applicable, the following three letters after the species name refer to the location of capture (IND: indoor; OUT: outdoor) and the sampling month (JUL: July; SEP: September; OCT: October) is indicated before the year

Several studies have reported different molecular approaches to identify the *An. coluzzii* and *An. gambiae* species. However, Santolomazza et al. [48] reported that these methods are not entirely interchangeable and some differences have been reported among results. For this purpose, to avoid biases that could affect the interpretation of our genetic analyses, a subset of specimens, were randomly sampled and identified according to the polymorphism of nearly 200 bp-long the Short Interspersed Elements (SINE200) in division 6 of the X-chromosome as described by Santolamazza et al. [49].

### Microsatellite genotyping

Fourteen microsatellite loci (Table 3) selected from the genetic map of *An. gambiae* published by Zheng et al. [50] were used for genotyping studies. These markers were located on chromosome 3 to avoid potential bias resulting from selective pressures associated with chromosomal inversions on chromosome 2 [26] and genomic island of divergence regions on the X chromosome [29]. Microsatellite markers were amplified for each specimen by multiplexed PCR using fluorescently labeled (PET, NED, FAM, VIC) forward primer in a final volume of 10 µl containing 5 µl of 2× PCR Master Mix (Applied Biosystems^®^), 1 µl of primer mix, 3 µl of RNase free water and 1 µl of genomic DNA. Four primer mixes were constituted according to the annealing temperature (Ta) of markers in a final volume of 400 µl, consisting of 12.5 pmol of each primer. Amplification reactions were performed with an initial denaturation step of 10 min at 94 °C followed by 25–30 cycles of 30 s at 94 °C, 30 s at Ta °C, 30 s at 72 °C, and then an extension step of 5 min at 72 °C. PCR products were mixed with Genescan-500 Liz size standard and deionized formamide (Applied Biosystem). Amplified fragments were separated by capillary electrophoresis in an automatic sequencer (ABI 3130xl Genetic Analyser) and the allele’s size scored using GeneMapper software (Applied Biosystem).

**Table 3:** Microsatellite markers used in this study, repeated pattern (RP) from Zheng et al. [50] and characteristics (allele size, AS; annealing temperature, Ta)

Marker locus	RP	Sequence primer flanking	AS (bp)	Ta (°C)	*An. coluzzii* (N = 119)		*An. gambiae* (N = 82)		*An. arabiensis* (N = 309)	
					Na	A	Na	A	Na	A
H59	(GT)9	5′ CCCCTATTAAACCCTGGACG 3′ 3′ TGTTGTTGCCCTGCGTTACC 5′	123	55	10	6.75 ± 1.71	7	6.00 ± 1.00	13	7.33 ± 1.50
H88	(GT)9	5′TGCGGCGGTAAAGCATCAAC 3′ 3′ CCGGTAACACTGCGCCGAC 5′	176	60	NPA	NPA	NPA	NPA	23	13.44 ± 3.05
H93	(GT)4 + 7	5′ TCCCCAGCTCACCCTTCAAG 3′ 3′ GGTTGCATGTTTGGATAGCG 5′	209	55	19	10.75 ± 3.86	20	12.67 ± 0.58	30	13.00 ± 3.81
H119	(GT)6	5′ GGTTGATGCTGAAGAGTGGG 3′ 3′ ATGCCAGCGGATACGATTCG 5′	174	55	17	12.25 ± 1.26	14	10.67 ± 0.58	10	6.89 ± 1.69
H128	(GT)21	5′ CGGGACGGCTAGATAAAGCG 3′ 3′ CCGGGCGACATAACCCACCC 5′	117	55	25	17.5 ± 3.42	24	16.67 ± 3.22	^b^	^b^
H242	(GT)8	5′TTCATTTCCACCGCAGCTGC 3′ 3′GGCGACACTCAATCCTTCC 5′	69	58	7	5.50 ± 1.00	7	5.67 ± 0.58	^c^	^c^
H249	(GT)15	5′ ATGTTCCGCACTTCCGACAC 3′ 3′ GCGAGCTACAACAATGGAGC 5′	129	60	22	13.50 ± 1.73	17	12.33 ± 1.16	27	12.67 ± 2.69
H312	(GT)10	5′ TAAACCATCAACCAGCCACC 3′ 3′ ACTGTGTGCGAGTCGGTTGG 5′	108	58	19	12.75 ± 1.50	16	13.00 ± 0.00	29	15.33 ± 4.79
H555	(GT)8	5′GCAGAGACACTTTCCGAAAC3′ 3′ TGTCAACCCACATTTTGCGC 5′	81	55	17	10.50 ± 3.00	15	9.67 ± 3.06	^b^	^b^
H577	(GT)16	5′ TTCAGCTTCAGGTTGGTCTC 3′ 3′ GGGTTTTTTGGCTGCGACTG 5′	113	55	13	8.50 ± 2.65	14	10.00 ± 1.00	22	10.78 ± 3.31
H746	(GT)14	5′ TGGGTTCGAAATTCGCCAAC 3′ 3′ GACGTGTGCACCCGTTGTG 5′	105	58	14	11.25 ± 1.26	13	10.67 ± 1.16	29	16.89 ± 2.57
H758	(GT)11	5′ TGATTTGCCAGTTCTGCCAG 3′ 3′ GTGATTGGAGTGGCTAGTGG 5′	105	60	^a^	^a^	^a^	^a^	11	5.11 ± 0.93
H812	(GT)10	5′CTGGCCCATTTTGCATATGC 3′ 3′ TGCTCCACCAAACCACATC 5′	131	55	^a^	^a^	^a^	^a^	15	10.00 ± 1.58
H817	(GT)8	5′ ACTGGTCCGTTGCTGCGCG 3′ 3′ ATGAGTGAATGGTGCGCTGG 5′	124	58	7	6.00 ± 0.82	6	5.00 ± 1.00	^b^	^b^
Mean					15.46 ± 5.87	10.48 ± 3.89	13.91 ± 5.59	10.21 ± 3.76	20.90 ± 7.56	11.14 ± 4.14

Parameters of polymorphism for *An. gambiae, An. coluzzii* and *An. arabiensis* populations are given: number of observed alleles (Na), mean number of alleles per locus (A), number of specimens used (N)

^a^Null alleles in *An. gambiae* and *An. coluzzii*; ^b^ null alleles in *An. arabiensis*, ^c^ monomorphic locus, not used in the study, *NPA* not properly amplified

### Data analysis

Microchecker 2.2.3 software developed by Van Oosterhout et al. [51] was initially used to examine possible genotyping errors due to null alleles. Genetic diversity was assessed per locus, per population, by estimating the number of observed alleles (Na), and by the mean number of alleles per locus (A) using Arlequin.3.5.1 [52]. The same software was used to test deviation from Hardy–Weinberg equilibrium (HWE) for each locus in each population and to estimate the observed heterozygosity (Ho) and expected heterozygosity (He). Statistical significance of HWE was assessed by the exact probability test available in Genepop 3.2 software [53] and the same software was used to estimate the inbreeding coefficient (Fis) and the linkage disequilibrium between each pair of loci in each population. A possibly significant heterozygosity excess (the signature of a bottleneck) was tested with the Bottleneck program [54] using two mutational models: an infinite allele model (IAM) and a stepwise mutation model (SMM). The probabilities for the sign tests (ps) for heterozygosity excess and for pw Wilcoxon test (two-tailed for heterozygote excess or deficiency) were calculated. Genetic differentiation between populations was assessed using pairwise estimates Fst values according to Weir and Cockerham, computed using Arlequin software [52]. Additionally, an assignment test implemented in the Geneclass 2 program [54] and developed by Paetkau et al. [55] was used for populations of each species to estimate the likelihood of an individual’s multilocus genotype being assigned to the population in which it has the highest likelihood of belonging, compared to the likelihood of being assigned to other populations. The test was thus used including all the individuals of both *An. coluzzii* and *An. gambiae*. This partition was used as a reference to assign hybrids to a most likely *An. coluzzii* or/and *An. gambiae* population using the Geneclass 2 program [54].

## Results

### Species composition

In the study 611 individuals were included, namely 136 *An. coluzzii,* 101 *An. gambiae*, 6 *An. coluzzii/An. gambiae* hybrids (2.5%) and 368 *An. arabiensis* (Table 1), according to IMP-PCR [47]. Note that the hybrid frequency was stable for 2006 and 2008 (3.2 and 3.03%), and decreased significantly after 2008 (1.2% in 2010). A subset of 497 individuals thus determined was also tested according to SINE200 [49]. The concordance between the species identification methods was 97.32% for *An. coluzzii,* 95.55% for *An. gambiae* and 99.65% for *An. arabiensis.* For the six *An. gambiae/An. coluzzii* hybrids only one was identified as a hybrid with the second method. Therefore, the species identified according to IMP-PCR which could discriminate more hybrids were analyzed with microsatellite markers. Hybrids individuals were then discarded for the general population genetic studies but not for the assignment test.

### Genetic structure of *Anopheles* populations

Fourteen microsatellites previously defined on *An. gambiae* were tested (Table 3). Polymorphism parameters for *An. gambiae*, *An. coluzzii* and *An. arabiensis* samples (all pooled populations) are given in Table 3. All microsatellites were polymorphic except H127, which was monomorphic in the three species, and H242 in *An. arabiensis*. Moreover, the locus H88 failed to amplify properly, particularly in *An. coluzzii* and *An. gambiae* individuals, even after optimization of PCR conditions. Thus, these loci were removed from further analyses in the concerned species. Moreover, Microchecker analysis performed on each *Anopheles* population showed the presence of null alleles at the H812 and H758 loci within *An. gambiae* and *An. coluzzii* populations, and H817, H128, and H555 in *An. arabiensis* populations (Table 3). Therefore, these loci were also discarded. A total of 11 polymorphic loci were analysed in *An. gambiae and An. coluzzii* populations and 10 in *An. arabiensis* populations.

For the sister species, the total number of observed alleles (Na) for the polymorphic locus ranged from 7 (H242 and H817) to 25 (H128) for *An. coluzzii* and 6 (H817) to 24 (H128) for *An. gambiae,* with a mean of 10.48 and 10.21 alleles per locus respectively (Table 3). The multilocus analysis performed for each population showed a mean of allele per locus (A) varying between 8.00 (AC10) and 12.45 (ACOUT06) in *An. coluzzii* populations and from 10.09 (AG06) to 10.36 (AGOUT10) in *An. gambiae* populations (Table 4). The observed heterozygosity ranged from 0.706 (ACIND06) to 0.742 (ACOUT06) in *An. coluzzii* populations and 0.690 (AGOUT10) to 0.725 (AG06) in *An. gambiae* populations. Significant heterozygote deficits from the Hardy–Weinberg equilibrium were observed in all populations. Positive Fis values were observed, ranging from 0.073 (ACOUT06) to 0.112 (AC08) in *An. coluzzii* populations, and 0.106 (AG06) to 0.131 (AGIND10) and 0.146 (AGOUT10) in *An. gambiae* populations, but were not significant according to the confidence interval (Table 4). The Hardy–Weinberg equilibrium tested in each population for each locus showed 17/44 and 17/33 significant departures from Hardy–Weinberg proportions in equilibrium within population of *An. coluzzii* and *An. gambiae,* respectively (Additional file 1: Table S1).

**Table 4:** Genetic variability for each population of *An. coluzzii* (AC), *An. gambiae* (AG), and *An. arabiensis* with: sample size (N), mean number of alleles for all loci (A), observed heterozygosity (Ho), expected heterozygosity (He), P value in Hardy–Weinberg equilibrium and inbreeding coefficient (Fis) and confidence interval (CI), probability for H excess or deficiency for the sign tests (p_s_) with, in brackets, the ratio of the number of loci with heterozygote excess to the number with heterozygote deficiency and for the one-tailed Wilcoxon test for H excess (p_w_) for the infinite allele model (IAM) and the stepwise mutation model (SMM)

Populations	N	A ± se	Ho ± se	He ± se	P value ± se	Fis CI 95%	IAM	SMM
ACIND06	22	9.455 ± 3.934	0.706 ± 0.202	0.785 ± 0.135	0.000 ± 0.000	0.102 (−0.004 to 0.160)	p_s_ = 0.305 (8/3) *p*_*w*_ = 0.027	p_s_ = 0.036 (3/8) p_w_ = 0.966
ACOUT06	57	12.455 ± 4.458	0.742 ± 0.113	0.800 ± 0.108	0.000 ± 0.000	0.073 (0.023 to 0.109)	*p*_*s*_ = 0.033 (10/1) *p*_*w*_ = 0.006	p_s_ = 0.00005 (0/11) p_w_ = 1.00000
AC08	25	10.909 ± 3.646	0.734 ± 0.105	0.824 ± 0.107	0.000 ± 0.000	0.112 (0.042 to 0.137)	p_s_ = 0.127 (9/2) *p*_*w*_ = 0.008	p_s_ = 0.033 (3/8) p_w_ = 0.949
AC10	15	8.000 ± 2.898	0.739 ± 0.150	0.810 ± 0.140	0.000 ± 0.000	0.090 (−0.017 to 0.106)	p_s_ = 0.130 (9/2) *p*_*w*_ = 0.034	p_s_ = 0.116 (4/7) p_w_ = 0.861
AG06	23	10.091 ± 4.110	0.725 ± 0.164	0.809 ± 0.095	0.000 ± 0.000	0.106 (0.037 to 0.126)	p_s_ = 0.118 (9/2) *p*_*w*_ = 0.034	p_s_ = 0.110 (4/7) p_w_ = 0.949
AGIND10	32	10.182 ± 3.125	0.693 ± 0.136	0.796 ± 0.116	0.000 ± 0.000	0.131 (0.061 to 0.175)	p_s_ = 0.130 (9/2) *p*_*w*_ = 0.034	p_s_ = 0.007 (2/9) p_w_ = 0.995
AGOUT10	27	10.364 ± 4.056	0.690 ± 0.156	0.806 ± 0.103	0.000 ± 0.000	0.146 (0.053 to 0.203)	*p*_*s*_ = 0.031 (10/1) *p*_*w*_ = 0.027	p_s_ = 0.031 (3/8) p_w_ = 0.938
AROUTSEP06	15	8.500 ± 3.240	0.560 ± 0.130	0.760 ± 0.137	0.000 ± 0.000	0.270 (0.134 to 0.326)	p_s_ = 0.361 (5/5) p_w_ = 0.500	p_s_ = 0.180 (4/6) p_w_ = 0.920
ARINDJUL08	44	14.000 ± 5.603	0.738 ± 0.125	0.805 ± 0.086	0.000 ± 0.000	0.085 (0.021 to 0.127)	p_s_ = 0.613 (6/4) p_w_ = 0.500	p_s_ = 0.0001 (0/10) p_w_ = 1.000
AROUTJUL08	72	14.400 ± 5.696	0.649 ± 0.097	0.749 ± 0.103	0.000 ± 0.000	0.135 (0.090 to 0.165)	p_s_ = 0.354 (5/5) p_w_ = 0.754	p_s_ = 0.00012 (0/10) p_w_ = 1.000
ARINDSEP08	33	11.800 ± 4.237	0.680 ± 0.091	0.780 ± 0.113	0.000 ± 0.000	0.131 (0.049 to 0.179)	p_s_ = 0.631 (6/4) p_w_ = 0.385	p_s_ = 0.00014 (0/10) p_w_ = 1.000
AROUTSEP08	22	8.500 ± 3.308	0.643 ± 0.092	0.749 ± 0.133	0.005 ± 0.002	0.145 (0.045 to 0.188)	p_s_ = 0.366 (7/3) p_w_ = 0.216	p_s_ = 0.171 (4/6) p_w_ = 0.984
ARINDSEP10	38	11.400 ± 4.088	0.619 ± 0.101	0.755 ± 0.114	0.000 ± 0.000	0.181 (0.121 to 0.214)	p_s_ = 0.362 (5/5) p_w_ = 0.423	p_s_ = 0.00015 (0/10) p_w_ = 1.000
AROUTSEP10	27	10.500 ± 3.923	0.692 ± 0.116	0.766 ± 0.117	0.000 ± 0.000	0.099 (0.020 to 0.138)	p_s_ = 0.358 (5/5) p_w_ = 0.461	p_s_ = 0.002 (1/9) p_w_ = 0.999
ARINDOCT10	19	9.400 ± 3.134	0.730 ± 0.112	0.756 ± 0.098	0.009 ± 0.003	0.035 (−0.065 to 0.081)	p_s_ = 0.366 (5/5) p_w_ = 0.839	p_s_ = 0.002 (1/9) p_w_ = 0.999
AROUTOCT10	39	11.700 ± 4.029	0.686 ± 0.06	0766 ± 0.104	0.000 ± 0.000	0.106 (0.046 to 0.135)	p_s_ = 0.609 (6/4) p_w_ = 0.423	p_s_ = 0.00016 (0/10) p_w_ = 0.991

Those in italic indicate only the significant P value for H excess

Concerning the *An. arabiensis* species, a higher polymorphism than for the two sister species is shown with a total number of alleles ranging from 10 (H119) to 30 (H93), with a mean value of 20.9 and a mean number of alleles per locus of 11.14 (Table 3). The multilocus study performed for each population showed the minimum mean number of alleles of all loci in AROUTSEP06 and AROUTSEP08 populations (8.50) and the maximum in ARINDJUL08 and AROUTJUL08 (14.000 and 14.400) (Table 4). The mean observed heterozygosis (Ho) in *An. arabiensis* samples ranged from 0.560 (AROUTSEP06) to 0.738 (ARINDJUL08). All the samples presented a deficit in heterozygotes from the Hardy–Weinberg equilibrium. Positive inbreeding coefficients (Fis) ranged from 0.036 to 0.27 but were not significant according to the confidence interval (Table 4). The Hardy-Weinberg equilibrium tested for each locus showed 40/90 significant departures from Hardy–Weinberg proportions in equilibrium (Additional file 1: Table S2).

### Genetic differentiation between populations

Estimates of pairwise Fst values were calculated between populations of each sister species (*An. coluzzii, An. gambiae),* and between populations of the two species (Table 5). Except for two *An. gambiae* populations (AGIND10 and AGOUT10), no significant differentiation within *An. coluzzii* or *An. gambiae* populations (P > 0.05) was observed, indicating for each species no genetic differentiation between populations sampled indoors and outdoors, and/or in different years, including before (2006), during (2008) and after (2010) the implementation LLINs. However, between *An. coluzzii* and *An. gambiae* populations a significant but low level of genetic differentiation is observed for 8 pairwise comparisons out of 12, with Fst values ranging from 0.008 to 0.025. *An. coluzzii* populations sampled in 2010 and outdoors in 2006 were genetically differentiated from all the *An. gambiae* populations whatever the year (2006, 2010) or the place of sampling (indoors or outdoors). Moreover, the *An. gambiae* outdoor population sampled in 2010 (AGOUT10) was also genetically differentiated from all the *An. coluzzii* population whatever the year or the place of sampling. No genetic differentiation is observed between the *An. coluzzii* population sampled indoors in 2006 (ACIND06) or in 2008 (AC08) and all the indoor *An. gambiae* populations, whatever the year (AGIND06, AGIND10).

**Table 5:** Genetic differentiation between pair of *An. coluzzii* and *An. gambiae* samples estimated by Fst values (below diagonal) of 11 loci, Fst P value ± standard error (above diagonal), and values in italic represent significant Fst values at 5%

Populations	ACIND06	ACOUT06	AC08	AC10	AGIND06	AGIND10	AGOUT10
ACIND06	0.000	0.449 ± 0.005	0.426 ± 0.006	0.386 ± 0.005	0.150 ± 0.004	0.081 ± 0.003	*0.007* *±* *0.001*
ACOUT06	0.001	0.000	0.155 ± 0.004	0.161 ± 0.004	*0.027* *±* *0.002*	*0.0004* *±* *0.0002*	*0.000* *±* *0.000*
AC08	0.003	0.004	0.000	0.308 ± 0.005	0.082 ± 0.003	0.091 ± 0.003	*0.007* *±* *0.001*
AC10	0.004	0.006	0.005	0.000	*0.013* *±* *0.001*	*0.000* *±* *0.000*	*0.003* *±* *0.001*
AGIND06	0.007	*0.008*	0.008	*0.016*	0.000	0.525 ± 0.006	0.436 ± 0.001
AGIND10	0.008	*0.011*	0.007	*0.025*	0.001	0.000	*0.05* *±* *0.002*
AGOUT10	*0.016*	*0.015*	*0.014*	*0.021*	0.003	*0.008*	0.000

In *An. arabiensis* populations seven pairwise comparisons out of 36 led to significant Fst values, ranging from 0.06 to 0.014 (Table 6). Furthermore, no genetic differentiation was observed between populations captured indoors and outdoors in the same month, except populations captured in July 2008 (ARINDJUL08/AROUTJUL08). Nevertheless, significant Fst values are observed between sample populations captured from 1 month to another and from 1 year to another.

**Table 6:** Genetic differentiation between pairs of *An. arabiensis* samples estimated by Fst values (below diagonal) of ten loci, Fst P value ± standard error (above diagonal), and values in italic represent significant Fst values at 5%

Populations	AROUTSEP06	ARINDJUL08	AROUTJUL08	ARINDSEP08	AROUTSEP08	ARINDSEP10	AROUTSEP10	ARINTOCT10	AROUTOCT10
AROUTSEP06	0.000	0.273 ± 0.004	0.267 ± 0.005	0.745 ± 0.004	0.794 ± 0.004	0.777 ± 0.004	0.825 ± 0.004	0.634 ± 0.005	0.245 ± 0.004
ARINDJUL08	0.005	0.000	*0.008* *±* *0.001*	*0.030* *±* *0.002*	0.197 ± 0.005	*0.0002* *±* *0.0001*	0.141 ± 0.003	0.295 ± 0.005	0.531 ± 0.006
AROUTJUL08	0.006	*0.007*	0.000	0.053 ± 0.002	0.480 ± 0.005	0.088 ± 0.003	0.846 ± 0.003	0.509 ± 0.006	*0.035* *±* *0.002*
ARINDSEP08	0.0002	*0.007*	0.006	0.000	0.883 ± 0.004	*0.015* *±* *0.001*	0.325 ± 0.005	0.379 ± 0.005	*0.031* *±* *0.002*
AROUTSEP08	0	0.005	0.001	−0.003	0.000	0.380 ± 0.005	0.808 ± 0.004	0.791 ± 0.004	0.228 ± 0.004
ARINDSEP10	0	*0.014*	0.005	*0.011*	0.004	0.00000	0.949 ± 0.002	0.617 ± 0.005	*0.004* *±* *0.001*
AROUTSEP10	0	0.005	0	0.003	0	0	0.000	0.856 ± 0.004	0.466 ± 0.005
ARINDOCT10	0.002	0.003	0.001	0.003	0	0.001	0	0.000	0.325 ± 0.005
AROUTOCT10	0.007	0.00053	*0.006*	*0.008*	0.004	*0.012*	0.001	0.003	0.000

The analysis of data using Bottleneck software showed that all the populations of *An. coluzzii* and *An. gambiae* have significant heterozygote deficit under the IAM model with the Wilcoxon test, while the sign test showed only two populations (ACOUT06 and AGOUT10) exhibiting a putative bottleneck. However, under this model, no significant heterozygote excess was detected for the *An. arabiensis* populations whatever the test used. Under the SMM model, no significant heterozygote excess was detected in populations whatever the species.

### Assignment test

The results of the assignment test using Geneclass 2 revealed that for *An. coluzzii* populations, less than a quarter of individuals (about 8–13.33%) are classified in their original population, except one population (ACOUT06). For *An. gambiae* populations, around half of the individuals were assigned to their original population except AG06 (21.74%) (Table 7). However, when all samples of *An. coluzzii* are pooled, 75.63% of individuals are correctly assigned to *An. coluzzii*, and a similar percentage is observed for *An. gambiae* (73.17%). The assignment test in hybrids showed that four individuals were highly assigned to *An. coluzzii* (percentage assignment >95%) while two were highly assigned to *An. gambiae* (Table 9). For *An. arabiensis* populations, 7.4–22% of individuals are assigned to their original population (Table 8).

**Table 7:** The proportion of correct assignment of individuals performed with Geneclass 2 in *An. coluzzii* and *An. gambiae* samples

	ACIND06	ACOUT06	AC08	AC10	AG06	AGIND10	AGOUT10
ACIND06	9.09	21.05	16	20	17.39	15.63	0
ACOUT06	22.73	36.84	24	20	13.04	6.25	0
AC08	18.18	7.02	8	26.67	0	6.25	7.40
AC10	13.64	10.53	20	13.33	4.35	0	7.40
AG06	18.18	14.04	12	6.67	21.74	9.38	18.52
AGIND10	13.64	10.53	12	0	26.09	40.63	22.22
AGOUT10	4.55	0	8	13.33	17.39	21.88	44.44
Total	22	57	25	15	23	32	27

**Table 8:** The proportion of correct assignment of individuals performed with Geneclass 2 in *An. arabiensis* samples

	AROUT SEP06	ARIND JUL08	AROUT JUL08	ARIND SEP08	AROUT SEP08	ARIND SEP10	AROUT SEP10	ARIND OCT10	AROUT OCT10
AROUTSEP06	13.33	13.64	5.56	9.09	13.64	7.89	11.11	10.53	0
ARINDJUL08	13.33	20.45	13.89	6.06	0	2.63	7.40	0	10.26
AROUTJUL08	6.67	9.09	22.22	18.18	13.64	15.79	3.70	10.53	2.56
ARINDSEP08	13.33	4.55	12.5	18.18	27.27	2.63	0	5.26	7.69
AROUTSEP08	6.67	15.91	11.11	15.15	13.64	7.89	22.22	26.32	15.39
ARINDSEP10	20	4.55	15.28	9.09	9.09	21.05	22.22	10.53	12.82
AROUTSEP10	13.33	4.55	9.72	6.06	18.18	13.16	7.40	10.53	12.82
ARINDOCT10	6.67	6.82	11.11	6.06	4.55	15.79	14.81	10.53	23.07
AROUTOCT10	6.67	20.45	11.11	12.12	0	13.16	11.11	15.79	15.38
Total	15	44	72	33	22	38	27	19	39

**Table 9:** The proportion of correct assignment of individuals performed with Geneclass 2 in all samples of *An. coluzzii*, *An. gambiae* and hybrids

	*An. coluzzii*	*An. gambiae*
*An. coluzzii*	75.63 (90)	26.83 (22)
*An. gambiae*	24.37 (29)	73.17 (60)
Hybrids	66.67 (4)	33.33 (2)
Total	123	84
Hybrid individuals	*An. coluzzii*	*An. gambiae*
HYBINDOCT06	99.795	0.205
HYBINDOCT06	0.118	99.882
HYBINDOCT06	100.000	0.000
HYBINDSEP06	96.176	3.824
HYBINDSEP08	99.919	0.081
HYBINDSEP10	6.292	93.708

The total number of the individuals tested is indicated in brackets. Details for hybrids assigned in *An. coluzzii* and *An. gambiae* populations are given in the table

## Discussion

The three major vectors of *An. gambiae* complex, *An. coluzzii, An. gambiae* and *An. arabiensis,* have an overlapping distribution in Dielmo as described in many parts of West Africa [5]. The aim of the present study was to elucidate whether implementation of LLINs in Dielmo has an impact on the genetic structure of these populations. Using microsatellite markers, the genetic structure of mosquito populations was analysed before (2006), during (2008) and after (2010) the implementation of LLINs in Dielmo. These microsatellites mapped throughout the chromosome 3 have been chosen to avoid confounding patterns of genetic structure associated to linked-markers on polymorphic inversions on chromosome 2 and putative genes of reproductive isolation on the X chromosome [26, 29]. The results of *An. coluzzii* and *An. gambiae* populations should be interpreted with caution due to the low population size of samples compared with those of *An. arabiensis,* and the resulting pooling of some samples. However, the observed heterozygosity is quite similar whatever the year and the species with significant deficits in heterozygotes in all populations. Various factors could explain the observed heterozygote deficit compared to the expected heterozygosity under HWE. A Wahlund effect due to sample bias cannot be discarded for some pooled samples, but genetic drift due to repetitive reduction in population size resulting from bottlenecks is likely, since all the *An. coluzzii* and *An. gambiae* populations exhibit heterozygote excess under the IAM model using the Wilcoxon test with Bottleneck software. This is congruent with entomological data from a previous study showing that after the implementation of LLINs in Dielmo, the relative abundance of *Anopheles* populations fluctuated substantially in our study area, with a dramatic decrease of *An. coluzzii* and *An. gambiae,* while *An. arabiensis* increased and remained the prevalent species [28]. However, no significant inbreeding is noticed (F_IS_ values), so at each generation, gene flow would be sufficient to prevent inbreeding. Between pairs of populations within each species no significant genetic structuration (F_ST_ values) was observed except the two *An. gambiae* populations sampled indoors and outdoors in 2010 after the implementation of LLINs in Dielmo. Between the sister species, the lack of genetic differentiation between some populations reveals the occurrence of a gene flow. Because the results of the assignation test using neutral markers fails to shown a clear pattern of hybridization (for hybrids, the assignation is not equal for the two species), results are in favour of introgressed individuals, with backcrossing of hybrids with parental species. The asymmetrical situation is observed with a higher proportion of hybrids assigned to *An. coluzzii,* in favour of the introgression of the genome of *An. coluzzii* by *An. gambiae*, except for the only one hybrid in 2010. From a methodological point of view, it should be noted that the IMP-PCR and SINE200 methods are quite equivalent for the species identification but the IMP-PCR method seems more acute to detect hybrids.

Previous studies in West and Central African countries where *An. coluzzii* and *An. gambiae* are frequently sympatric reported a strong deficit of hybrids in nature that was below 1%, resulting in reproductive isolation between both species [56, 57]. However, hybrids rates ranged from 7% in Gambia [57] and over 20% in Guinea Bissau [7, 8, 36, 58]. In the study, the figure of 2.5% of hybrids is in agreement with a previous study performed in Senegal [42]. In Guinea Bissau high genetic divergence was found between *An. coluzzii* and *An. gambiae* (Fst value = 0.348), but 60% of field-collected hybrids were backcrossed [8]. Indeed, Lee et al. [11] reported that the assortative mating between *An. coluzzii* and *An. gambiae* periodically breaks down, explaining high levels of hybridization in various sites in West Africa lying outside coastal areas (5.2–96.9%). Additionally in the laboratory, Diabaté et al. [59] found that F1 hybrids are fully fertile and viable.

In Benin, the occurrence of the Kdr mutation in *An. coluzzii* populations was attributed to an introgression process involving *An. gambiae,* which already presented the *kdr* mutation [60]. In Mali Norris et al. [14] showed before the implementation of ITNs and IRS, a breakdown of assortative mating resulting in high levels of hybrids in 2006 and the introgression of the Kdr mutation in *An. coluzzii* populations. This led to an increase in the frequency of selectively advantaged backcrossed individuals after the widespread introduction of ITNs. In our study, between *An. coluzzii* and *An. gambiae* populations, significant gene flow was found in 2006 and 2008, but for 2010, all the Fst values were significant. Thus, as for Mali populations, there was a breakdown of assortative mating resulting in hybrids in the same year 2006, but the introduction of LLINs had a negative impact on hybridization frequency, which is corroborated by the decrease in the number of hybrids observed in the data after 2008. This study suggests decreased mating success of hybrids or ecological maladaptation, but also a lesser probability of mating between species due to a decrease in *An. coluzzii* population size. The lack of *An. coluzzii* could explain the occurrence in 2010 of a unique hybrid assigned to *An. gambiae*, probably resulting from hybrid backcrossing with the most populous species, *An. gambiae*. Then, the genetic divergence could be maintained between the two species despite gene flow, which is consistent the speciation-with-gene flow model as proposed by Turner et al. [29].

Regarding the sibling species *An. arabiensis,* results suggested some spatial and temporal structuration between populations, especially in 2008, of the seven significant F_ST_ pairwise comparisons, six involved in 2008 collected populations. If *An. arabiensis,* the most widespread member of the *An. gambiae* complex was described generally to be genetically less structured compared to the sibling species *An. coluzzii* and *An. gambiae* populations, some studies demonstrated genetic distinct subpopulations [37, 39]. The level of genetic differentiation in *An. arabiensis* populations varies significantly according to geographical distance, as demonstrated in Senegalese and Indian Ocean island populations [40] or in Sudanese to Mozambican populations [61], but also on a local scale in a village in Tanzania due to ecological diversification [39]. Polymorphic inversions were found at differential frequencies in West Africa than in East Africa *Anopheles* populations [62, 63] and between outdoor and indoor populations [26]. Inversions were involved in local adaptation, with the selection of co-adapted genes affecting behaviour activities [64, 65]. Marsden et al. [66] suggested that chromosomal inversions contribute to population structure in *An. arabiensis*. This species, although generally considered to be a less efficient vector, was a major malaria vector in Dielmo due to its high human biting rates [67]. This species presents behaviour plasticity according its feeding activities, resting places and also to its great tendency to survive during the dry season [68]. After the implementation of LLINs in 2008 to Dielmo, a reconfiguration of vector abundance was noted, a large decrease in population size was observed in *An. coluzzii* and *An. gambiae,* but an expansion of *An. arabiensis* [28]. The lack of bottleneck effects nor significant inbreeding in populations are in adequacy with large population size. A higher genetic diversity was observed in July 2008 compared with 2006 for both indoor and outdoor populations. This could result from the migration of individuals coming from genetically divergent populations. Indeed, an entomological study conducted in villages around the Dielmo area showed that most of the *Anopheles* populations in these areas belong to *An. arabiensis* [69]. The higher genetic diversity coincides with the date of LLIN implementation. A diversifying selection could be envisaged, favouring individuals with genotypes diverging from the mean genotype of the population. Various scenarios could be proposed, such as the occurrence of various resistance genes conferring individual advantage and then their selection, while non-resistant individuals were eliminated. The selection of individuals with divergent behaviour in biting activities could be envisaged, since it was demonstrated in Dielmo for another vector, *Anopheles funestus,* where a shift to diurnal feeding, essentially after the introduction of LLINs, was observed. This highlights the need to explore the evolution of the genetic basis of behavioural traits in *Anopheles* populations in the context of vector control of malaria transmission.

## Conclusions

Several studies in Africa carried out after the implementation of interventions strategies have focused on the survival, the status of insecticide resistance and behavioural biting changes of *Anophele*s and on vector composition. However, little information is available about the genetic structure of the main malaria vectors of the *An. gambiae* complex involved in Senegal, which is particularly worrying in the context of high coverage of insecticide-treated bed nets in the malaria control programme. In this study, the potential effects of the implementation of LLINs on the genetic structure of *An. gambiae, An. coluzzii* and *An. arabiensis* were demonstrated. According to the species, the effect after the implementation of LLINs in Dielmo is differentiated. The sister species *An. coluzzii* and *An. gambiae* populations regularly experienced bottleneck, but without significant inbreeding. Since a breakdown of assortative mating resulted in hybrids, the introduction of ITNs had a negative impact on hybridization frequency. As pointed by Marsden et al. [8] the occurrence of a various level of hybrids across West Africa probably results from a “geographic mosaic of reproductive isolation” but this phenomenon could have considerable implications for transgenic control strategies.

Regarding the sibling species *An. arabiensis,* the study suggested some spatial and temporal structuration between populations, especially in 2008, coinciding with the date of LLINs implementation, which could result from diversifying selection favouring the expansion of this species. Taking into account that vector control is the cornerstone for reducing the burden of malaria disease and that current tools are mainly based on insecticide-treated bed nets, multidisciplinary studies combining epidemiology, ecology, and population genetics are needed to define successful management of malaria vector control.

## Additional file

**Additional file 1: Table S1.** Genetic variability for each locus within *An. coluzzii* and *An. gambiae* populations, observed heterozygosity (Ho), expected heterozygosity (He), P value in Hardy–Weinberg equilibrium and inbreeding coefficients (Fis). In bold: locus in Hardy–Weinberg disequilibrium. **Table S2.** Genetic variability for each locus within *An. arabiensis* populations, observed heterozygosity (Ho), expected heterozygosity (He), P value in Hardy–Weinberg equilibrium and inbreeding coefficients (Fis). In bold: locus in Hardy–Weinberg disequilibrium.

## Abbreviations

LLINs: long-lasting insecticidal treated bed nets
IRS: indoor residual spraying
HLC: human landing catches
HWE: Hardy–Weinberg equilibrium
IAM: infinite allele model
SMM: stepwise mutation model
