# Supplementary material for: The implementation of long-lasting insecticidal bed nets has differential effects on the genetic structure of the African malaria vectors in the Anopheles gambiae complex in Dielmo, Senegal
Source: Malar J. 2017 Aug 15;16:337. doi: 10.1186/s12936-017-1992-8 (PMC5558778; doi:10.1186/s12936-017-1992-8)
Supplement: Supplementary file 1 — Additional file 1: Table S1. Genetic variability for each locus within An. coluzzii and An. gambiae populations, observed heterozygosity (Ho), expected heterozygosity (He), P value in Hardy–Weinberg equilibrium and inbreeding coefficients (Fis). In bold: locus in Hardy–Weinberg disequilibrium. Table S2. Genetic variability for each locus within An. arabiensis populations, observed heterozygosity (Ho), expected heterozygosity (He), P value in Hardy–Weinberg equilibrium and inbreeding coefficients (Fis). In bold: locus in Hardy–Weinberg disequilibrium. [file 12936_2017_1992_MOESM1_ESM.doc]

**Table S1**. Genetic variability for each locus within *An. coluzzii* and *An. gambiae* populations, observed heterozygosity (Ho), expected heterozygosity (He), P-value in Hardy Weinberg equilibrium and inbreeding coefficients (Fis). In bold: locus in Hardy Weinberg disequilibrium

| **Populations** |  | **H242** | **H746** | **H817** | **H249** | **H119** | **H577** | **H59** | **H128** | **H312** | **H555** | **H93** | **Mean** |
| --- | --- | --- | --- | --- | --- | --- | --- | --- | --- | --- | --- | --- | --- |
| **ACIND06** | Ho | 0.273 | 0.864 | 0.500 | 0.857 | 0.909 | 0.590 | 0.818 | 0.818 | 0.864 | 0.727 | 0.545 | 0.706±0.202 |
|  | He | 0.481 | 0.873 | 0.627 | 0.856 | 0.904 | 0.672 | 0.810 | 0.902 | 0.890 | 0.811 | 0.807 | 0.785±0.135 |
|  | P-value | **0.017** | 0.175±0.023 | **0.014± 0.002** | 0.487± 0.037 | 0.610±0.030 | 0.143± 0.0160 | 0.383± 0.016 | **0.032± 0.020** | 0.120± 0.021 | 0.202± 0.017 | **0.0004± 0.0004** | 0.000± 0.000 |
|  | Fis | 0.439 | 0.011 | 0.206 | -0.001 | -0.006 | 0.124 | -0.013 | 0.095 | 0.030 | 0.105 | 0.329 | 0.102 |
| **ACOUT06** | Ho | 0.509 | 0.732 | 0.667 | 0.786 | 0.893 | 0.632 | 0.860 | 0.860 | 0.719 | 0.789 | 0.719 | 0.742±0.113 |
|  | He | 0.601 | 0.876 | 0.709 | 0.842 | 0.878 | 0.621 | 0.797 | 0.920 | 0.870 | 0.833 | 0.857 | 0.800±0.108 |
|  | P-value | **0.0005±0.0002** | **0.013± 0.006** | **0.004± 0.001** | 0.232 ± 0.038 | 0.710± 0.035 | 0.717±0.031 | 0.678±0.026 | 0.352 ± 0.061 | **0.000± 0.000** | 0.258 ± 0.022 | **0.0002±0.0002** | 0.000± 0.000 |
|  | Fis | 0.154 | 0.165 | 0.061 | 0.067 | -0.016 | -0.017 | -0.080 | 0.066 | 0.174 | 0.053 | 0.164 | 0.073 |
| **AC08** | Ho | 0.560 | 0.760 | 0.625 | 0.875 | 0.875 | 0.800 | 0.708 | 0.680 | 0.667 | 0.840 | 0.680 | 0.734±0.105 |
|  | He | 0.588 | 0.856 | 0.728 | 0.915 | 0.889 | 0.754 | 0.733 | 0.921 | 0.896 | 0.891 | 0.891 | 0.824±0.107 |
|  | P-value | 0.121±0.007 | **0.013 ± 0.006** | **0.042± 0.005** | 0.058± 0.014 | 0.567±0.024 | 0.703±0.038 | 0.448 ± 0.010 | **0.002± 0.001** | **0.020 ± 0.013** | 0.110± 0.031 | **0.003±0.002** | 0.000± 0.000 |
|  | Fis | 0.048 | 0.115 | 0.144 | 0.045 | 0.200 | -0.062 | 0.035 | 0.266 | 0.260 | 0.058 | 0.241 | 0.112 |
| **AC10** | Ho | 0.533 | 0.667 | 0.667 | 0.867 | 0.733 | 0.533 | 0.800 | 1.000 | 0.933 | 0.667 | 0.733 | 0.739±0.150 |
|  | He | 0.54416 | 0.88247 | 0.77792 | 0.90325 | 0.88050 | 0.79189 | 0.73312 | 0.91349 | 0.90714 | 0.88445 | 0.90321 | 0.810±0.140 |
|  | P-value | **0.005±0.001** | **0.003±0.002** | 0.359± 0.016 | 0.109± 0.020 | 0.051 ± 0.016 | 1.000± 0.000 | 0.557± 0.013 | 1.000 ± 0.000 | 0.860± 0.018 | **0.006± 0.002** | 0.324±0.016 | 0.000±0.000 |
|  | Fis | 0.228 | 0.247 | 0.120 | 0.050 | -0.041 | -0.179 | 0.012 | -0.055 | -0.045 | 0.224 | 0.102 | 0.090 |
| **AG06** | Ho | 0.739 | 0.696 | 0.435 | 0.864 | 0.909 | 0.522 | 0.870 | 0.783 | 0.870 | 0.522 | 0.773 | 0.725±0.163 |
|  | He | 0.684 | 0.847 | 0.681 | 0.911 | 0.84 | 0.664 | 0.793 | 0.927 | 0.882 | 0.797 | 0.841 | 0.809±0.095 |
|  | P-value | 0.798±0.013 | 0.060±0.009 | **0.001± 0.001** | 0.390±0.036 | 0.796±0.013 | **0.010±0.003** | 0.884± 0.004 | **0.010± 0.01** | 0.214± 0.029 | **0.001 ± 0.001** | 0.290± 0.031 | 0.000± 0.000 |
|  | Fis | -0.082 | 0.182 | 0.367 | 0.053 | 0.323 | 0.218 | -0.099 | 0.158 | 0.015 | 0.351 | 0.083 | 0.106 |
| **AGINT10** | Ho | 0.438 | 0.688 | 0.656 | 0.875 | 0.600 | 0.567 | 0.800 | 0.750 | 0.844 | 0.812 | 0.594 | 0.693±0.136 |
|  | He | 0.529 | 0.849 | 0.698 | 0.865 | 0.881 | 0.670 | 0.777 | 0.886 | 0.878 | 0.853 | 0.867 | 0.796±0.116 |
|  | P-value | **0.047±0.004** | **0.002±0.001** | 0.302±0.010 | 0.656±0.022 | **0.000±0.000** | **0.018±0.008** | 0.727±0.018 | **0.038±0.01** | **0.043±0.017** | 0.361±0.034 | **0.000±0.000** | 0.000±0.000 |
|  | Fis | 0.176 | 0.192 | 0.061 | -0.012 | 0.205 | 0.157 | -0.030 | 0.155 | 0.040 | 0.048 | 0.3187 | 0.131 |
| **AGOUT10** | Ho | 0.481 | 0.815 | 0.481 | 0.925 | 0.704 | 0.481 | 0.815 | 0.667 | 0.815 | 0.630 | 0.778 | 0.690±0.156 |
|  | He | 0.68418 | 0.86271 | 0.65989 | 0.88418 | 0.88192 | 0.59774 | 0.76271 | 0.93164 | 0.84463 | 0.81977 | 0.85593 | 0.806±0.103 |
|  | P-value | **0.012±0.002** | **0.044± 0.010** | 0.065 | 0.665± 0.037 | **0.000±0.000** | **0.005±0.003** | 0.826±0.010 | **0.000±0.000** | 0.232±0.036 | **0.008±0.004** | 0.056±0.015 | 0.000± 0.000 |
|  | Fis | 0.300 | 0.076 | 0.288 | -0.043 | 0.204 | 0.228 | -0.057 | 0.287 | 0.040 | 0.247 | 0.089 | 0.146 |
| Mean | He | 0.607 ± 0.082 | 0.866 ± 0.014 | 0.696 ± 0.041 | 0.884 ± 0.030 | 0.888± 0.014 | 0.637 ± 0.092 | 0.784 ± 0.027 | 0.919 ± 0.021 | 0.879 ± 0.017 | 0.838 ± 0.031 | 0.847 ± 0.030 | 0.804± 0.115 |
|  | P-value | 0.000±0.000 | 0.000±0.000 | 0.000±0.000 | 0.096±0.017 | 0.001±0.0006 | 0.009±0.004 | 0.913± 0.009 | 0.000±0.000 | 0.000±0.000 | 0.000±0.000 | 0.000±0.000 | 0.000±0.000 |
|  | Fis | 0.167 | 0.142 | 0.155 | 0.026 | 0.085 | 0.072 | -0.043 | 0.140 | 0.095 | 0.130 | 0.193 | 0.110 |

**Table S2**. Genetic variability for each locus within *An.arabienis* populations, observed heterozygosity (Ho), expected heterozygosity (He), P-value in Hardy Weinberg equilibrium and inbreeding coefficients (Fis). In bold: locus in Hardy Weinberg disequilibrium

| Populations |  | **H746** | **H812** | **H249** | **H758** | **H88** | **H119** | **H577** | **H59** | **H312** | **H93** | **Mean** |
| --- | --- | --- | --- | --- | --- | --- | --- | --- | --- | --- | --- | --- |
| **AROUTSEP06** | Ho | 0.533 | 0.733 | 0.533 | 0.400 | 0.400 | 0.600 | 0.400 | 0.600 | 0.733 | 0.667 | 0.560 ± 0.130 |
|  | He | 0.931 | 0.871 | 0.834 | 0.660 | 0.837 | 0.625 | 0.687 | 0.501 | 0.883 | 0.775 | 0.760 ± 0.137 |
|  | P-value | **0.000±0.000** | 0.066± 0.012 | **0.000±0.000** | **0.002** | **0.000±0.000** | 0.534 | **0.003±0.002** | 1.000± 0.000 | 0.107±0.015 | 0.223±0.044 | 0.000 ±0.000 |
|  | Fis | 0.436 | 0.163 | 0.369 | 0.402 | 0.530 | 0.041 | 0.247 | -0.201 | 0.174 | 0.144 | 0.270 |
| **AROUTJUIL08** | Ho | 0.523 | 0.705 | 0.884 | 0.705 | 0.698 | 0.727 | 0.614 | 0.750 | 0.955 | 0.818 | 0.738 ± 0.125 |
|  | He | 0.927 | 0.857 | 0.844 | 0.695 | 0.803 | 0.719 | 0.699 | 0.766 | 0.926 | 0.815 | 0.805 ± 0.086 |
|  | P-value | **0.000 ±0.000** | **0.037 ± 0.006** | 0.413 ± 0.057 | 0.548 ± 0.011 | **0.000 ± 0.000** | **0.033 ± 0.006** | 0.126 ± 0.037 | 0.110 ± 0.014 | 0.674 ± 0.068 | 0.335 ± 0.046 | 0.000 ±0.000 |
|  | Fis | 0.439 | 0.180 | -0.047 | -0.014 | 0.133 | -0.011 | 0.123 | 0.021 | -0.032 | -0.004 | 0.085 |
| **ARINDJUIL8** | Ho | 0.549 | 0.611 | 0.750 | 0.556 | 0.551 | 0.571 | 0.690 | 0.739 | 0.818 | 0.656 | 0.649 ± 0.097 |
|  | He | 0.889 | 0.802 | 0.812 | 0.567 | 0.693 | 0.641 | 0.705 | 0.743 | 0.885 | 0.756 | 0.749 ± 0.103 |
|  | P-value | **0.000 ± 0.000** | **0.001 ± 0.001** | 0.120 ± 0.034 | **0.002 ± 0.001** | **0.000 ± 0.000** | **0.003 ± 0.001** | 0.491 ± 0.052 | 0.356 ± 0.025 | 0.074 ± 0.038 | 0.011 ± 0.011 | 0.000 ±0.000 |
|  | Fis | 0.384 | 0.239 | 0.077 | 0.020 | 0.208 | 0.109 | 0.021 | 0.005 | 0.076 | 0.133 | 0.135 |
| **ARINDSEP08** | Ho | 0.531 | 0.788 | 0.719 | 0.576 | 0.688 | 0.636 | 0.697 | 0.667 | 0.839 | 0.656 | 0.680 ± 0.091 |
|  | He | 0.914 | 0.890 | 0.846 | 0.614 | 0.811 | 0.658 | 0.642 | 0.726 | 0.900 | 0.802 | 0.780 ± 0.113 |
|  | P-value | **0.000±0.000** | **0.000±0.000** | **0.046±0.008** | 0.261±0.019 | **0.005±0.004** | 0.249±0.020 | 0.138±0.014 | **0.019±0.006** | 0.326±0.051 | 0.065±0.020 | 0.000 ± 0.000 |
|  | Fis | 0.423 | 0.117 | 0.153 | 0.063 | 0.154 | 0.033 | -0.087 | 0.083 | 0.069 | 0.184 | 0.131 |
| **AROUTSEP08** | Ho | 0.619 | 0.667 | 0.619 | 0.545 | 0.524 | 0.600 | 0.750 | 0.700 | 0.818 | 0.591 | 0.643 ± 0.092 |
|  | He | 0.931 | 0.864 | 0.863 | 0.528 | 0.727 | 0.708 | 0.694 | 0.604 | 0.888 | 0.682 | 0.749 ± 0.133 |
|  | P-value | **0.000±0.000** | **0.003±0.001** | **0.001±0.0001** | 0.672 | 0.061±0.011 | 0.295±0.012 | 0.857±0.026 | 0.955±0.003 | **0.014±0.006** | **0.037±0.008** | 0.005 ± 0.000 |
|  | Fis | 0.341 | 0.233 | 0.288 | -0.033 | 0.285 | 0.157 | -0.084 | -0.164 | 0.080 | 0.136 | 0.145 |
| **ARINDSEP10** | Ho | 0.595 | 0.737 | 0.579 | 0.474 | 0.605 | 0.632 | 0.676 | 0.552 | 0.816 | 0.526 | 0.619 ± 0.101 |
|  | He | 0.883 | 0.805 | 0.791 | 0.573 | 0.843 | 0.636 | 0.782 | 0.620 | 0.901 | 0.711 | 0.755 ± 0.114 |
|  | P-value | **0.000±0.000** | **0.005±0.003** | **0.000±0.000** | 0.145±0.015 | **0.000±0.000** | 0.565±0.024 | **0.032±0.007** | 0.364±0.028 | 0.058±0.022 | **0.006±0.002** | 0.000 ±0.000 |
|  | Fis | 0.330 | 0.086 | 0.270 | 0.175 | 0.285 | 0.008 | 0.137 | 0.110 | 0.096 | 0.262 | 0.181 |
| **AROUTSEP10** | Ho | 0.444 | 0.741 | 0.852 | 0.630 | 0.667 | 0.667 | 0.619 | 0.750 | 0.741 | 0.815 | 0.692 ± 0.116 |
|  | He | 0.929 | 0.786 | 0.857 | 0.558 | 0.846 | 0.657 | 0.721 | 0.656 | 0.871 | 0.787 | 0.766 ± 0.117 |
|  | P-value | **0.000±0.000** | 0.052±0.052 | 0.095±0.018 | 0.919 | **0.001±0.001** | 0.605±0.023 | 0.222±0.031 | 0.970±0.005 | **0.016±0.005** | 0.427±0.065 | 0.000 ± 0.000 |
|  | Fis | 0.527 | 0.059 | 0.007 | -0.130 | 0.215 | -0.015 | 0.145 | -0.147 | 0.152 | -0.036 | 0.099 |
| **ARINDOCT10** | Ho | 0.789 | 0.947 | 0.737 | 0.579 | 0.737 | 0.632 | 0.778 | 0.737 | 0.789 | 0.579 | 0.730 ± 0.112 |
|  | He | 0.893 | 0.868 | 0.767 | 0.637 | 0.782 | 0.651 | 0.667 | 0.698 | 0.883 | 0.713 | 0.756 ± 0.098 |
|  | P-value | 0.051±0.019 | 0.683±0.021 | 0.044±0.014 | 0.328±0.013 | 0.090±0.014 | 0.408±0.014 | 0.924±0.014 | 0.861±0.014 | 0.294±0.030 | **0.005±0.004** | 0.009 ± 0.003 |
|  | Fis | 0.119 | -0.095 | 0.040 | 0.094 | 0.060 | 0.031 | -0.172 | -0.057 | 0.109 | 0.192 | 0.035 |
| **AROUTOCT10** | Ho | 0.658 | 0.744 | 0.658 | 0.605 | 0.737 | 0.590 | 0.718 | 0.692 | 0.703 | 0.757 | 0.686 ± 0.057 |
|  | He | 0.920 | 0.842 | 0.788 | 0.571 | 0.779 | 0.704 | 0.655 | 0.726 | 0.863 | 0.816 | 0.766 ± 0.104 |
|  | P-value | **0.000±0.000** | **0.028±0.009** | **0.002±0.002** | 0.815±0.009 | 0.233±0.036 | **0.001±0.001** | 0.537±0.049 | 0.530±0.017 | **0.0001±0.0001** | **0.002±0.002** | 0.000 ±0.000 |
|  | Fis | 0.287 | 0.119 | 0.167 | -0.062 | 0.054 | 0.164 | -0.097 | 0.047 | 0.188 | 0.073 | 0.106 |
| **Mean** | He | 0.913 ± 0.019 | 0.843 ± 0.037 | 0.823 ± 0.034 | 0.600 ± 0.054 | 0.791± 0.053 | 0.667 ± 0.035 | 0.694 ± 0.041 | 0.671 ± 0.084 | 0.889 ± 0.018 | 0.762 ± 0.050 | 0.765± 0.111 |
|  | P-value | 0.000±0.000 | 0.000±0.000 | 0.000±0.000 | 0.0002±0.0001 | 0.000±0.000 | 0.000±0.000 | 0.118±0.020 | 0.236±0.017 | 0.000±0.000 | 0.000±0.000 | 0.000 ±0.000 |
|  | Fis | 0.374 | 0.142 | 0.123 | 0.038 | 0.196 | 0.063 | 0.038 | 0.001 | 0.089 | 0.113 | 0.120 |
